# Arteriovenous Fistula after Anatomic All-Inside Anterior Cruciate Ligament Reconstruction

**DOI:** 10.1155/2017/1034018

**Published:** 2017-11-26

**Authors:** Mathijs C. H. W. Fuchs, Martijn Dietvorst, Roel Vaes, Maarten Loos, Matthijs P. Somford, Rob P. A. Janssen

**Affiliations:** ^1^Department of Orthopaedic Surgery and Traumatology, Maxima Medisch Centrum, Postbus 7777, 5500 MB Veldhoven, Netherlands; ^2^Department of Vascular Surgery, Maxima Medisch Centrum, Postbus 7777, 5500 MB Veldhoven, Netherlands; ^3^Department of Orthopaedic Surgery and Traumatology, Rijnstate Hospital, Postbus 9555, 6800 TA Arnhem, Netherlands

## Abstract

We present the first case of an arteriovenous fistula after an all-inside anterior cruciate ligament (ACL) reconstruction. A seventeen-year-old boy had an uneventful ACL reconstruction. Four weeks after surgery, the patient was seen with a pulsating swelling at the lateral distal upper leg. Vascular consultation led to the diagnosis of pseudoaneurysm and arteriovenous fistula of the lateral superior genicular artery. Most likely, fistula is caused by the stab incision for preparation of the femoral tunnel, and no anatomical cause was found. The clinical presentation, previous cases of arteriovenous fistula after arthroscopic ACL reconstruction, possible causes, and management are discussed.

## 1. Introduction

The overall incidence of ACL injury is 78 per 100,000 persons [1]. The group between 15 and 39 years of age shows an incidence of 85–91 in 100,000 people and could be described as the group at risk [2]. Reconstruction of the ACL ranks number 6 of the most performed orthopaedic operations [3]. Vascular complications after ACL reconstructions are rare but can cause serious morbidity and potential mortality [4]. Vascular complications may occur after various methods of reconstruction and fixation [5]. Since 2013, we have switched to the all-inside ACL reconstruction technique (Arthrex, München). This technique is a modification of the full tibial tunnel technique in the form of a tibial socket, resulting in less pain and less traumatic drilling by using the FlipCutter technique and stab incisions [6].

In this report, we present the first case of an arteriovenous fistula after an all-inside anterior cruciate ligament reconstruction.

## 2. Case Report

A seventeen-year-old boy presented at the outpatient clinic. Previous medical history reveals the diagnosis of anterior cruciate ligament rupture after a noncontact trauma of the right knee at age twelve. A conservative brace treatment was initiated, and surgery was postponed till closure of the growth plates.

**Figure 1:**
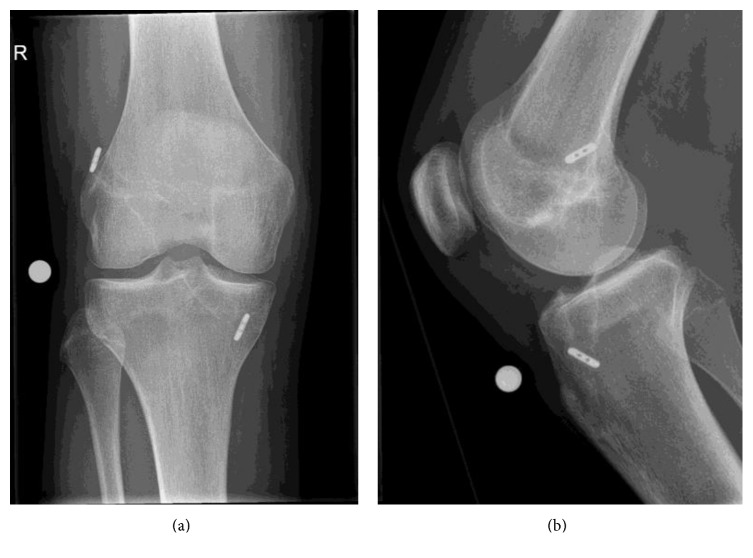
(a) and (b) Postoperative X-rays after arthroscopic ACL reconstruction.

Four weeks prior to the outpatient visit, he underwent an all-inside ACL reconstruction with hamstring tendon autograft with TightRope fixations [6]. The procedure was uneventful, and the patient was discharged the day after surgery. The X-ray showed adequate tunnel and fixation of the graft one day after the surgery (Figures 1 and 1). Routine follow-up after two weeks showed a normal postoperative knee: adequate wound healing and slight swelling of the joint with a range of motion 0°–110° without signs of neurovascular complications.

Reasons for the unscheduled outpatient visit at 4 weeks were complaints of a new swelling 10 days previously on the lateral distal upper leg. The patient felt slight pain at the site of the swelling at the end of the day although he was not limited during daily living or rehabilitation. Physical examination showed a painless, pulsating swelling, sized two by two centimetres. Location was nearby the stab incision made for the femoral fixation of the ACL graft. There was a slight swelling of the knee, range of motion was 110° to 0°, and the lower leg was neurovascular intact.

**Figure 2:**
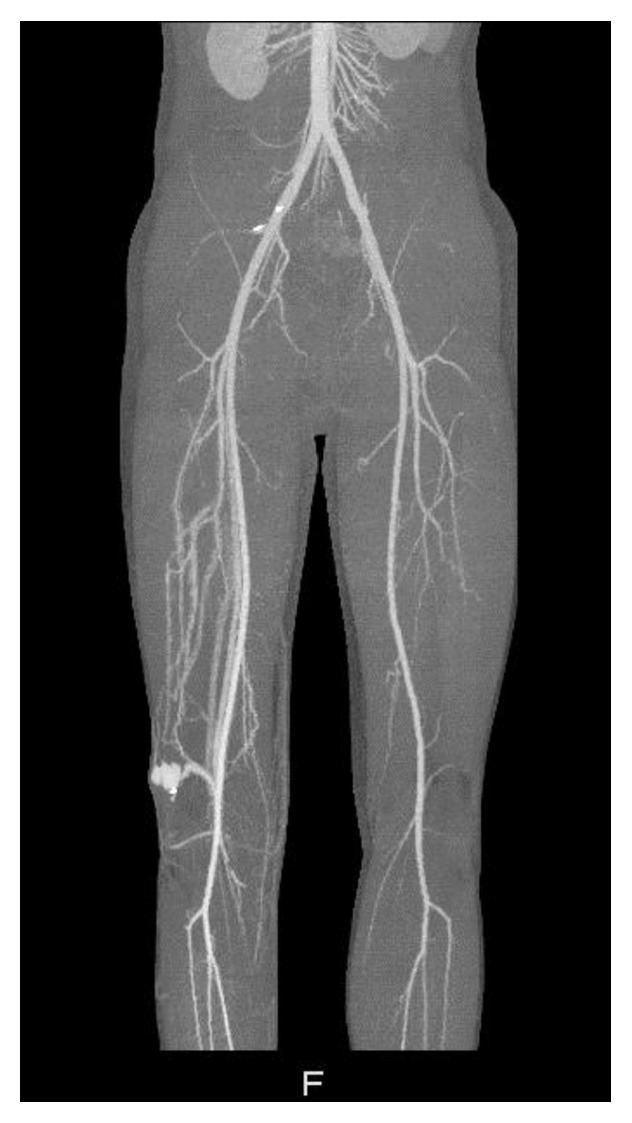
CT angiography showing the pseudoaneurysm and AV fistula lateral of the right knee.

CT angiography demonstrated a pseudoaneurysm, sized 27 × 26 × 26 millimetres (Figure 2). The origin was the arteria genicular lateralis superior with a venous backflow to the vena poplitea, suggesting an arteriovenous (AV) fistula. Extensive consultation with vascular surgeon and interventional radiologist led to surgical exploration of the pseudoaneurysm and AV fistula. Both thrombin injection of the aneurysm and coiling of the AV fistula were considered hazardous because of the risk of central venous occlusion or embolism creation.

**Figure 3:**
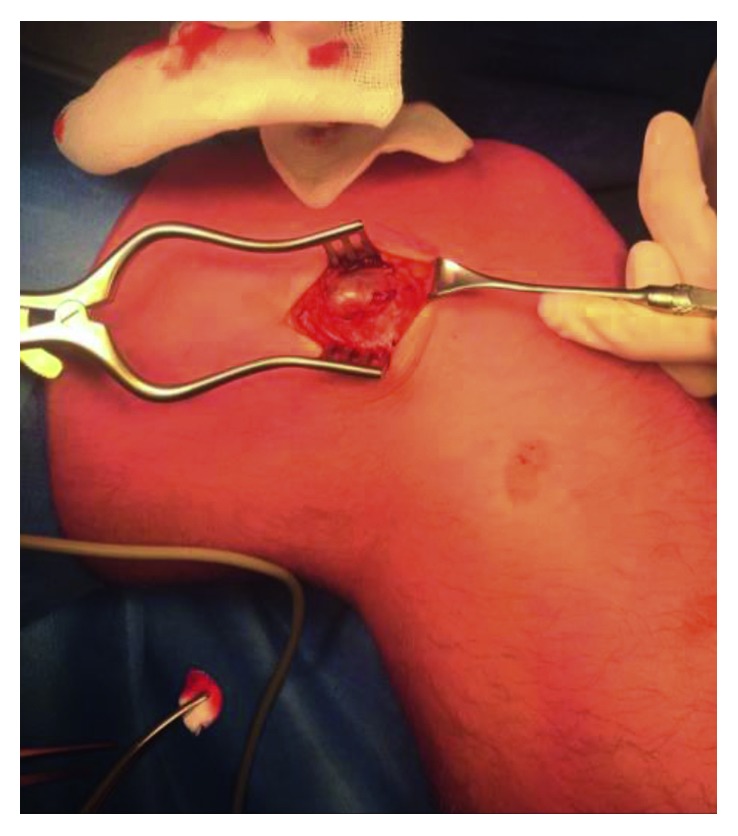
Surgical exploration of the pseudoaneurysm.

**Figure 4:**
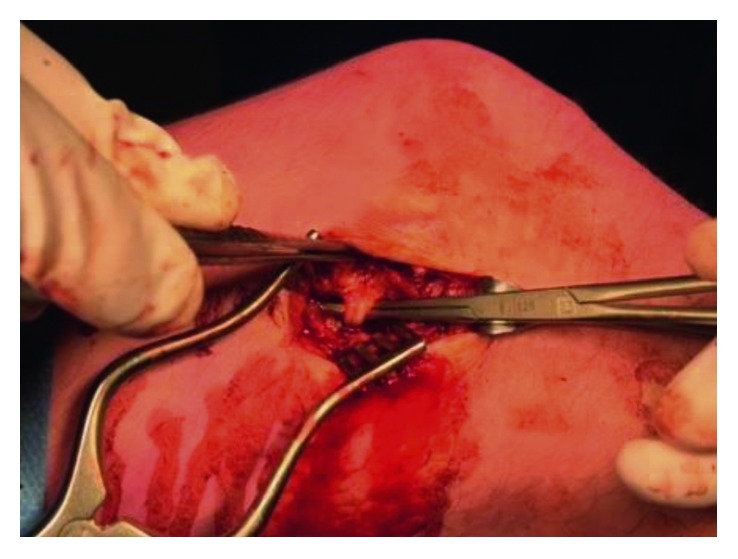
Identification of the accompanying genicular artery and vein that were ligated.

During surgery, the aneurysm was isolated (Figure 3) and excised while both the afferent and efferent artery and vein were ligated (Figure 4). The TightRope fixation could be identified directly underneath the aneurysm and was neatly covered by subcutaneous tissue during wound closure.

Two weeks after surgery, all complaints were resolved and the wound was healed. The routine ACL reconstruction rehabilitation restarted. At the follow-up after three months, the knee was painless and the patient did not experience any limitation of his knee. Physical examination showed absent swelling, range of motion 140° to 0°, and adequate ligament stability with an anterior drawer and Lachman test of 0–2 mm and an absent pivot shift.

## 3. Discussion

This case demonstrated a patient with an iatrogenic pseudoaneurysm with AV fistula after an all-inside anterior cruciate ligament reconstruction.

Arterial complications after ACL reconstruction are rare. The incidence of arterial lesions after ACL reconstruction is only described by Janssen et al. [4].

They analysed their consecutive series of 1961 arthroscopic ACL reconstructions between 1998 and 2014 and found an incidence of 0.15%. Two previous case reports have described AV fistulae after arthroscopic ACL reconstruction.

Carr and Jansson [7] described a 31-year-old male with an ACL reconstruction using an allograft Achilles tendon, fixed with a washer and bone plug. Seven weeks after surgery, a pulsating mass was discovered at his medial superior portal site. Examination by general surgeon confirmed the diagnosis of an AV fistula. Surgical exploration and excision of the mass were performed.

Keçeci et al. [8] described a 50-year-old woman with an ACL reconstruction using a bone patellar bone autograft. Soon after the operation, the authors report complaints of swelling and pain in the posterior knee. The authors do not specify the time interval of appearance after the ACL surgery. Vascular consultation was done due to complaints of a swollen foot and signs of vascular ischemia. The diagnosis of AV fistula was made by Doppler ultrasound investigation. Cause of the AV fistula was malposition of the femoral interference screw. After a revision of the ACL reconstruction and disconnection of the AV fistula, the vascular complaints disappeared. Further recovery was uneventful with 24-month follow-up.

AV fistulas are abnormal connections between the arterial and venous system that bypass the normal anatomic capillary beds. In the lower extremity, the cause is mostly iatrogenic or traumatic, for example, puncture, gunshots, and stab wounds [9]. AV fistulas originate due to laceration of an adjacent vein in conjunction with an artery. Initially, patients do not have complaints of the fistula. Onset of symptoms ranges from two days to several months [10]. Symptoms suggestive of AV fistula include abnormal sensation or palpable thrill, swelling, fatigue of the leg and signs of extremity ischemia can appear [9, 11].

**Figure 5:**
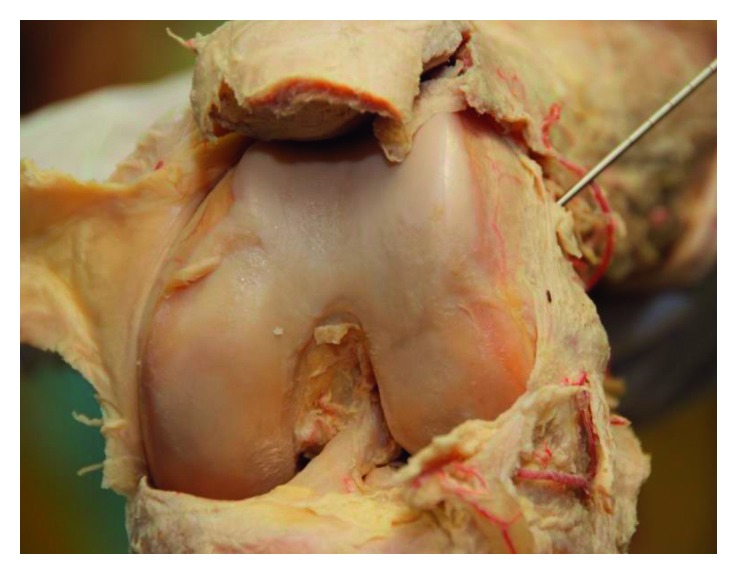
Position graft fixation outlined with the metal wand, cranial to the lateral superior genicular artery in red.

The most probable cause for the AV fistula in the present case was a laceration of the lateral superior genicular artery by stab incision for the femoral graft fixation. The position of this incision is determined by a guiding instrument to reconstruct the anatomical femoral footprint of the ACL (Figure 5). The incision is made approximately 1 centimetre anterior to the iliotibial tract and 2.5 centimetres proximal of the lateral femoral condyle. The superior genicular arteries branch from the popliteal artery and curve around the condyles anteriorly. The lateral superior genicular artery is located in a “triangle,” consisting of the vastus lateralis anteriorly, the short head of the biceps femoris posteriorly, and the femur condyle inferiorly. The artery subdivides in a superficial and deep branch inferior to the tendon of the biceps femoris. Both branches form anastomoses [12].

Diagnostic tests of choice to discover an AV fistula are duplex ultrasonography or computed tomographic angiography [13]. Small AV fistulas regress completely when thrombosis occurs [14]. Treatment is indicated for a patient who develops symptoms and can be surgical, ultrasound-guided compression, or by percutaneous endovascular techniques [14]. The prognosis for AV fistulas following repair is excellent [15].

In our opinion, this was an unavoidable incident. Due to anatomical vascular alterations, modification of the surgical technique does not guarantee prevention of this rare complication. This report illustrates the necessary awareness and possibility of early treatment for AV fistula after ACL reconstruction.

## 4. Conclusion

Vascular complications after ACL reconstruction are rare and may also occur in minimal invasive ACL reconstructions. Symptoms may vary and onset may be days to months after surgery. A good clinical and diagnostic follow-up is recommended in case of persistent vague symptoms after ACL reconstruction. Prognosis is excellent if AV fistula is recognized and treated early.
